# Urolithin A in Health and Diseases: Prospects for Parkinson’s Disease Management

**DOI:** 10.3390/antiox12071479

**Published:** 2023-07-24

**Authors:** Olga Wojciechowska, Małgorzata Kujawska

**Affiliations:** Department of Toxicology, Poznan University of Medical Sciences, Dojazd 30, 60-631 Poznań, Poland; 71464@student.ump.edu.pl

**Keywords:** urolithin A, ellagitannins, Parkinson’s disease, gut microbiota, neuroprotection, mitochondria

## Abstract

Parkinson’s disease (PD) is a chronic and progressive neurodegenerative disorder characterized by a complex pathophysiology and a range of symptoms. The prevalence increases with age, putting the ageing population at risk. Disease management includes the improvement of symptoms, the comfort of the patient’s life, and palliative care. As there is currently no cure, growing evidence points towards the beneficial role of polyphenols on neurodegeneration. Numerous studies indicate the health benefits of the family of urolithins, especially urolithin A (UA). UA is a bacterial metabolite produced by dietary ellagitannins and ellagic acid. An expanding body of literature explores the involvement of the compound in mitochondrial health, and its anti-inflammatory, anti-oxidant, and anti-apoptotic properties. The review organizes the existing knowledge on the role of UA in health and diseases, emphasizing neurodegenerative diseases, especially PD. We gathered data on the potential neuroprotective effect in in vivo and in vitro models. We discussed the possible mechanisms of action of the compound and related health benefits to give a broader perspective of potential applications of UA in neuroprotective strategies. Moreover, we projected the future directions of applying UA in PD management.

## 1. Introduction

Neurodegenerative disorders, including primarily Alzheimer’s disease (AD) and Parkinson’s disease (PD), represent a great threat to the ageing society. PD alone affects up to 3% of individuals over 65 years old and its prevalence is expected to increase by 50% by 2030 [1]. PD is a chronic human neurodegenerative disorder characterized by motor dysfunction. The disorder is described as a significant loss of dopaminergic neurons. A characteristic feature is the presence of Lewy bodies, caused mainly by the accumulation and aggregation of the amyloid formation of the α-synuclein protein, which spreads in the brain over time [2]. A substantial body of human and experimental evidence supports a prion-like mechanism of α-synuclein transmission that explains a slow and progressive spread of the pathology between brain regions in PD [3]. The treatment for PD is symptomatic, and is focused on improving motor symptoms and the comfort of the patient’s life. Palliative care is also a part of disease management, as no cure is currently available [4].

Meanwhile, there is ongoing research and growing evidence of the beneficial role of dietary polyphenols on the neurodegenerative process. The neuroprotective role is related to antioxidant, anti-inflammatory, and anti-apoptotic properties [5]. Ellagitannins and ellagic acid are polyphenols that occur naturally in pomegranates, strawberries, raspberries, walnuts, and almonds. The compounds are substrates of colonic bacteria’s transformation to urolithins. Urolithins are a family of microbial metabolites with potent biological activity [6]. However, the appropriate colon bacteria composition determines the ability to metabolize the specific polyphenols to urolithins. The production of urolithins depends on the age, health status, and dietary intake of the host [7]. Even though there are other types of urolithins, urolithin A (UA) is the most abundant and widely studied due to its benefits in health and disease [8]. 

In vivo studies on animal models explore the potential favorable effects of UA on ageing, muscle dysfunctions, cardiovascular disease, inflammatory bowel diseases (IBD), cancer, metabolic disorders, and brain health [8,9,10,11,12,13]. Regarding the latter, administration of the compound has been assessed mainly in AD and ischemic neuronal injury resulting in improved cognition, reduced neuroinflammation, neuronal loss, tau phosphorylation, and amyloid plaques [14,15,16,17]. Despite the promising research related to the cases mentioned above, there is still a gap in experimental in vivo models of PD. 

Taking into consideration the issues mentioned above, this review aims to organize the existing knowledge and explore the future directions of the role of UA in PD management. The review emphasizes the analysis of the studies focused on the potential neuroprotective effect of this bacteria-derived natural compound, especially in the context of PD. As the data are limited, we also presented possible mechanisms of the action and related other health benefits of UA to support its potential of neuroprotective activity, and to give a broader perspective of potential applications of this compound in PD. Moreover, we discussed a few studies on the antiparkinsonian effects of pomegranate juice treatment which was associated with the presence of UA.

## 2. Urolithin A Production and Mechanism of Action

### 2.1. Urolithins Metabolic Pathways

Urolithins, a class of organic compounds containing benzo-coumarin scaffolds, are metabolites of ellagitannins and ellagic acid produced by the gut microbiota. These polyphenols are found in plant products, mainly in pomegranates, berries, walnuts, and almonds, but also in some tropical fruits, medicinal plants, and herbal teas [7,18]. The bioavailability of ellagitannin is very low; however, their absorption may be increased by the co-intake of dietary fructooligosaccharides. The concentration of the compounds in the blood serum can also be modified by their dosage, duration of administration, post-intake time, and type of diet [7,19]. In the intestines, ellagitannins go through extensive metabolism by the bacteria [20]. The individual microbial composition determines the ability of the microbiota to convert natural polyphenols to urolithins [21]. It is noteworthy that the formation of urolithins also declines with age. Even though studies were conducted to identify the genera responsible for ellagitannins and ellagic acid conversion to urolithins, specific bacteria are still unknown [7,8].

**Figure 1:**
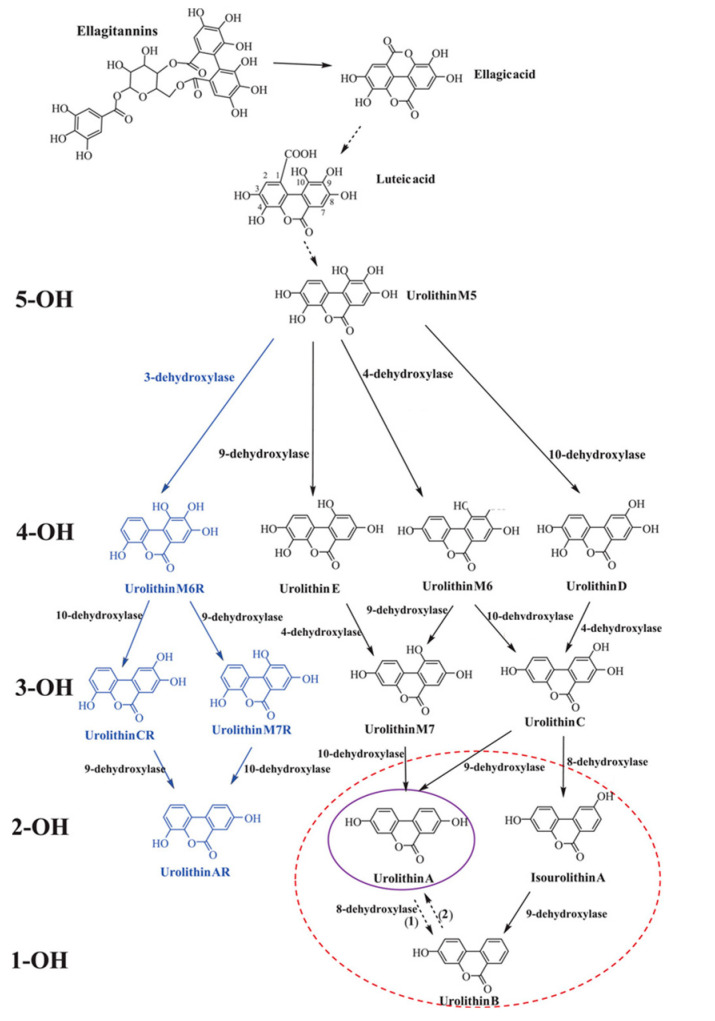
Catabolic pathway of ellagitannins and ellagic acid to urolithins. 5-OH, 4-OH, 3-OH, 2-OH, and 1-OH refer to the number of hydroxyl groups for each urolithin group—penta-, tetra-, tri-, di- and monohydroxy urolithins, respectively. The blue font refers to new urolithins generated by a bacterial 3-dehydroxylase. The purple and red circles designate the final urolithins produced in UM-A and UM-B, respectively. Uro-AR can be found in both metabotypes. Adapted from [7].

The catabolic pathway of ellagitannins and ellagic acid to urolithins includes lactone-ring cleavage, decarboxylation, and dehydroxylation reactions. Firstly, ellagitannins are hydrolyzed to ellagic acid by bacterial enzyme tannases. The extensive metabolism results in the creation of pentahydroxy-urolithin (urolithin M5), tetrahydroxy-urolithins (urolithin D, urolithin E, and urolithin M6), trihydroxy-urolithins (urolithins C and urolithin M7), dihydroxy-urolithins (urolithin A and isourolithin A), and monohydroxy-urolithin (urolithin B) [7,20]. In recent years, four more urolithins (M6R, M7R, CR, and AR) have been detected in human urine and/or fecal samples [22] (Figure 1). Urolithins A and B are the most abundant products of the pathway; however, UA is the most extensively studied [8]. After the absorption of UA into the bloodstream, the metabolite goes through the process of phase I and II metabolisms, including methylation and conjugation, with glucuronide and sulfate conjugates being the predominant compounds. The role and biological activity of UA conjugates are not yet fully known; nevertheless, in vitro studies suggest that their activity might be lower in comparison with UA [23]. Urolithins can concentrate in human biological fluids, including blood plasma (0.003–5.2 µM), urine (up to 50 µM), and breast milk (8.5–176.9 nM) [24]. Human and animal model studies report tissue and organ distributions of the compounds, including adipose tissue, brain, skeletal muscles, mammary tissue, kidneys, lungs, bladder, bone marrow, and organs along the gastrointestinal tract [25,26,27,28,29].

Intestinal conversion of urolithin precursors requires a specific gut microbiome, yet it is not entirely identified. It is believed that only 40% of individuals could naturally convert the polyphenolic precursors to UA [30]. Three metabotypes associated with the final urolithin products have been proposed: metabotype A (with only UA produced), metabotype B (UA, urolithin B and isourolithin A produced), and metabotype 0 (urolithin non-producers) [31]. The percentage of individuals unable to generate urolithins across ages 5 to 90 years is predicted to persist at 10%, and it can be affected by the state of dysbiosis [30]. Therefore, dietary supplementation is proposed to omit the requirement of the specific gut bacteria composition. The administration of UA is proposed to be an answer for urolithin non-producers, which could allow for the exploration of its health benefits [32].

### 2.2. Mechanism of Action

#### 2.2.1. Mitophagy and Mitochondrial Functions

Engagement in mitochondrial homeostasis is observed to be the coherent point in the in vivo model studies based on different types of animals and humans. One of the key modulators in the regulation of mitochondrial health is mitophagy. Mitophagy relates to the elimination of dysfunctional mitochondria in a selective autophagy process [33]. The process elevates the quality of the cellular mitochondria pool and is related to the creation of new organelles. The impairment of the process is related to ageing and several age-related diseases. The inhibition of mitophagy impacts mitochondrial homeostasis and could accumulate damaged organelles, resulting in cell damage [34]. 

UA’s beneficial role is related to the activation of several molecular pathways of mitophagy. In Caenorhabditis elegans, the administration of UA resulted in increased expression of genes responsible for mitophagy and autophagy [15,29,35]. The enhancement of the process in nematodes resulted in decreased accumulation of damaged organelles and increased mitochondrial biogenesis [29,35]. The involvement of the nuclear factor erythroid-derived 2-like 2 (Nrf2) homolog—skn-1 transcription factor was indicated to coordinate the regulation of mitophagy and mitochondrial biogenesis. The UA-induced mitophagy in worms contributed to maintaining mitochondrial respiratory capacity during ageing, related to extended lifespan and increased mobility. The robust mitochondria were able to sustain energy needs related to shifting respiration from complex I to complex II, as demonstrated in mammalian cells. In aged rodents, supplementation of UA also promoted mitophagy in the muscle of both young and old animals, accompanied by increased transcripts of mitophagy genes and ubiquitinated mitochondrial proteins in muscle tissues [29]. Similar outcomes were observed in models of Duchenne muscular dystrophy (DMD). In dystrophic worms, UA increased both mitochondrial quality and content, protecting against disease-associated muscle fiber degeneration and, as a result, increased motility of the dystrophic worms was observed. UA induced mitophagy-related genes and increased mitophagy flux in both human myoblasts from MDM patients and muscle tissues from dystrophic mice. Accordingly, enhanced mitophagy in DMD mice resulted in greater skeletal muscle respiratory capacity and improved cell regeneration and recovery [35]. Moreover, in a mouse model of AD, UA administration stabilized PTEN-induced kinase 1 (PINK1), reporting the protective role of mitophagy in neurons and microglia [15]. PI3K/Akt/mTOR-regulated autophagy was demonstrated to be involved in improving pancreatic β-cell function after UA administration in a mouse model of type 2 diabetes [36]. 

#### 2.2.2. Anti-Inflammatory Activity

A substantial body of research revealed the anti-inflammatory activity of UA. As some diseases, including those related to ageing, are associated with chronic, low-grade inflammation, UA’s ability to modulate inflammatory response is especially investigated [7]. Several animal studies explored the anti-inflammatory mechanism, including the modulation of genes, proteins, and signaling molecules. UA administration downregulated inflammatory cytokines/chemokine and immune cells in mice with cisplatin-induced nephrotoxicity [37] and resulted in reduced levels of the endoplasmic reticulum stress (ERS) markers, phosphorylated mitogen-activated protein kinase 8 and pro-inflammatory interleukins in obese mice [27]. Importantly, in mice fed a high-fat diet (HFD), UA promoted M2 polarization of peritoneal macrophages, thereby mediating attenuated inflammation [27]. In mouse models of colitis, UA administration led to a decrease in the same serum inflammatory markers and colonic neutrophil infiltration attributed to inhibited myeloperoxidase (MPO) activity. These effects were accompanied by enhanced gut barrier integrity [38]. Similarly, the improvement of the inflammation-induced intestinal barrier damage through modulation of the tight junction proteins’ expression was reported; however, epithelial cell models were employed [39]. Moreover, plasma IL-1β and TNF-α decreased, while IL-10 increased in diabetic mice after the administration of UA [36]. Also, UA treatment resulted in a decrease in neuroinflammation, reducing the levels of cerebral TNF-α and IL-6 and increasing IL-10 in AD mice accompanied by attenuated gliosis [15,16]. The restoration of neuronal mitophagy was indicated to enhance the phagocytic efficiency of microglia [15]. UA inhibited the activation of dendritic cells and microglia in the central nervous system (CNS) and the migration of pathogenic T cells from the periphery to the CNS in an experimental autoimmune encephalomyelitis (EAE) animal model [40]. The anti-inflammatory ability of UA might also be related to the suppression of phosphatidylinositol 3-kinase (PI3-K)/Akt/NF-κB and JNK/AP-1 signaling pathways, the abolishment of which resulted in the suppression of pro-inflammatory mediator production in lipopolysaccharide (LPS)-stimulated RAW264 macrophages [41]. In colitis and EAE mice, the anti-inflammatory effects were connected to the direct targeting of the aryl hydrocarbon receptor (AhR) and modulating the signaling pathways contributing to the inhibition of Th17 differentiation and the activation of dendritic cells and to a reduction in the inflammation barrier functions, respectively [38,40].

#### 2.2.3. Antioxidant Activity

Another possible mechanism of action is the beneficial role of the compound against oxidative stress. Studies have explored several processes that may correlate with the reduction of oxidative stress markers [7,8,12]. Pretreatment with UA or its natural precursors before oxidative stress exposure was reported to impact the performance of the antioxidant defense system [26,37]. The presence of UA upon administration of pomegranate juice, a rich source of ellagitannins, was associated with the increased activity of catalase (CAT), glutathione peroxidase (GPx), and glutathione S-transferase (GST), and a reduced level of glutathione (GSH) in the brains of rotenone-challenged rats [26]. Moreover, decreased lipid peroxidation accompanied by elevated activity of the mitochondrial enzyme protecting against oxidative stress—aldehyde dehydrogenase—was demonstrated [26]. Similar antioxidant effects were obtained in the cisplatin-induced nephrotoxicity model in mice [37]. UA administration protected against the cisplatin-induced depletion of the renal GSH pool, the inhibition of GPx and superoxide dismutase (SOD) activity, and a decrease in the gene expression of NADPH oxidase 2, which was the main source of oxidative stress in this model. As a result, declined lipid peroxidation and protein nitration were observed [37]. Similarly, UA enhanced the hepatic expression of SOD1 and SOD2 genes in obese mice [27] and augmented the pancreas’ GSH level, protecting against lipid peroxidation and diabetic mice [36]. In neuro-2a (N2a) cells subjected to oxidative stress, UA not only enhanced the cellular antioxidant mechanism attributed to increased CAT, SOD, glutathione reductase (GR), and GPx activity, but also inhibited oxidizing enzymes contributing to reactive oxygen species (ROS) production and directly scavenged radicals [42]. Moreover, UA was demonstrated to efficiently reduce the formation of advanced glycation end products, which interact with neuron cell surface receptors, triggering oxidative stress and inflammation, subsequently leading to neuronal dysfunction [43,44]. 

Beneficial effects of UA, including antioxidant activity, are believed to be mediated through the activation of the Nrf2/Kelch-like ECH-associated protein 1 (Keap1) signaling pathway [45]. Nrf2 is the transcription factor regulating the expression of antioxidant proteins engaged in the protection of cells and tissues against oxidative damage [7]. UA supplementation was reported to induce the expression of the pathway [38,46].

The available literature supports the use of UA to modulate oxidative stress and alleviate cell damage through different mechanisms [12]. As many diseases, including age-related disorders, are connected with the decreased activity of antioxidant enzymes and increased oxidative damage due to excessive ROS production, UA might be a promising active agent [42]. However, the antioxidative-related health benefits of the compound need further investigation, especially given earlier contradictory in silico findings on its inhibitory effect on GR, SOD, CAT, GST, and GPx by interfering with their active catalytic sites [47].

#### 2.2.4. Apoptosis-Modulating Activity

Interest has been generated in the ability of UA to regulate apoptosis. A recent review of the literature on this topic found that UA administration can affect both pro- and anti-apoptotic pathways with related anticancer or cell-loss-protecting effects, respectively [7]. 

The compound inhibited the pro-apoptotic pathway in both in vivo and in vitro models [16,37,48,49,50]. UA administration to cisplatin-injected mice attenuated the activity of renal caspase 3 activity and DNA fragmentation [37]. The inhibition of renal oxidative stress and apoptotic signaling in the cisplatin-induced acute kidney injury (AKI) mice model, accompanied by normalization of miRNA (miR-192-5p and miR-140-5p) implicated in AKI, resulted in improved survival rates [48]. Interestingly, in the APP/PS1 mouse model of AD, UA inhibited neuronal apoptosis in the cortex and hippocampus and enhanced neurogenesis at the same time [16]. In human neuroblastoma SH-SY5Y cells, UA treatment decreased oxidative-stress-induced apoptosis by preventing caspase-3 and 9 activation [49]. Similarly, UA decreased oxidative-stress-induced apoptosis in SK-N-MC cells by inhibiting the mitochondrial-related apoptosis pathway [50]. 

UA administration could also promote apoptosis. A growing body of literature suggests that UA is an anti-cancerogenic agent due to the modulation of the expression of genes, proteins, and signaling pathways involved in apoptosis induction [7,13,20]. In colorectal cancer cells, UA administration elevated the expression of the pro-apoptotic proteins (p53 and p21) and reduced anti-apoptotic protein expression (Bcl-2) [51]. Similarly, UA increased p53 and p21 protein expression in prostate cancer cells, inducing apoptosis [52]. Also, UA suppressed the viability of hepatocellular carcinoma HepG2.2.15 cells due to the activation of caspase-dependent apoptotic signaling by targeting the Lin28a/let-7a axis [53]. 

The induction of cell deaths and expression of relevant proteins is believed to depend on the cell type [8,12]. This suggests that UA is a promising compound with many potential biological activities. However, more research is needed to establish the specific actions in the different physiopathological settings [7,8]. 

The discussed data on the biological activities of UA has been gathered and presented below (Table 1).

**Figure 2:**
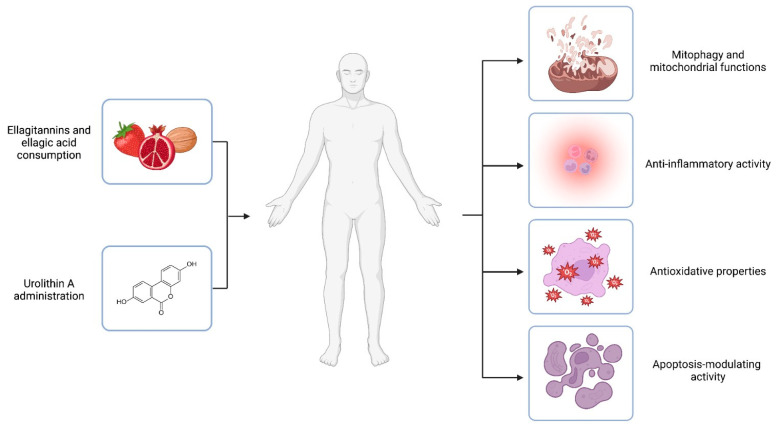
Urolithin A mechanisms of action. Ellagitannins and ellagic acid are polyphenols that occur naturally in dietary products like pomegranates, berries, and nuts. The compounds are substrates of colonic bacteria transformed into urolithins. However, it is estimated that only 40% of individuals could naturally convert the polyphenolic precursors to UA. Thus, UA administration is proposed as an answer for urolithin non-producers. In vivo and in vitro experiments suggest the health-promoting activity of UA. The effects of the compound are related to different mechanisms of action, including engagement in mitochondrial function and the process of mitophagy, inflammation, oxidative stress, and the modulation of the apoptosis process. Created with BioRender.com.

The contribution of these mechanisms (Figure 2) to health and diseases requires further study to understand the impact of UA in these areas.

## 3. Urolithin A in Health and Diseases

The impact of UA has been observed in a variety of different health conditions, including in in vivo and in vitro models. The research provides evidence of anti-inflammatory, antiapoptotic, and antioxidant properties, as well as cardioprotective and neuroprotective effects of the metabolite [8]. Many studies describe its beneficial effects in aging, muscle dysfunction, IBD, the nervous system, cardiovascular diseases, metabolic dysfunctions, and cancer [7]. Despite the promising in vivo results, the direct evidence of the health advantages and mechanisms of action of UA are still open to dispute.

The research on the properties attributed to UA exposure or administration, including animal models, increased in the last decade. Experiments show improvement in muscle strength, mobility, and exercise performance both in natural ageing [29,54,55] and regenerative ability in a DMD mice model [35]. The lifespan extension following UA treatment was dependent on the improvement of mitochondrial function [29,35], mitophagy, and autophagy [55], and the upregulation of angiogenic pathways via the Sirt1-PGC-1α mechanism [54] (Table 1 and Table 2). Furthermore, randomized, placebo-controlled trials including middle-aged [11] and older adults [56] came to similar conclusions. Human research reported that UA oral supplementation was safe and well-tolerated. In both studies, the intervention resulted in improved mitochondrial health, decreased levels of plasma markers of metabolism disorders (acylcarnitines), and systemic inflammation, including C-reactive protein and pro-inflammatory cytokines, as well as a significant enhancement in muscle endurance and strength [11,56] (Table 2). 

Meanwhile, the cardioprotective effects of UA include a decrease in risk markers, diastolic cardiac function, and atherosclerotic lesions [57,58,59]. Cardioprotection might also be related to improvement in metabolic dysfunctions, including obesity [27,61], diabetes, and insulin resistance [36,62]. UA administration was responsible for improved insulin sensitivity, decreased triglyceride accumulation, enhanced thermogenesis in brown adipose tissue, and the induction of white adipose tissue browning in obese mice. In the type 2 diabetic mice model, reduced levels of fasting glucose, glycated haemoglobin, interleukins, free fatty acids in serum, and significantly improved insulin-mediated glucose-lowering effects were observed after UA supplementation [36,62]. In healthy volunteers, UA intake enhanced vascular endothelial function that correlated with the alteration of their individual gut microbiota composition [60] (Table 2).

UA also presented anticancer properties, including antiproliferation, apoptosis promotion, and anti-angiogenesis. UA-inhibited cell proliferation in both androgen receptor-positive and androgen receptor-negative cells in prostate cancer [63] due to the synergistic action of p38-MAPK activation and suppression of Wnt/β-catenin signaling in HepG2 cells [64] and decreased snail protein expression and activity in lung cancer cells [65]. However, significant attention was drawn to its activity in colon cancer [20]. Chemopreventive properties included a dose-dependent anti-clonogenic effect through the increase in the senescence-associated β-galactosidase activity [66] and reducing the glycolytic potential via the p53/TIGAR axis [67]. In colorectal cancer cells, UA enhanced autophagy and apoptosis and suppressed cell cycle progression and DNA synthesis [68] (Table 2). Moreover, enhancing the gut barrier integrity caused by the UA administration may result in potential protective activities against colitis [10]. Prevention of colitis could be through the activation of pathways responsible for the upregulation of epithelial tight junction proteins [38] or protection against immune abnormalities [10] (Table 1).

Urolithin A was demonstrated to be safe, bioavailable, and well-tolerated in the in vivo animal models but also in human experiments. The variety of potential health benefits tended to be related to the activation of mitophagy, improvement of mitochondrial homeostasis, and reduction of inflammation [75]. However, research is focused mostly on tissue- or disease-specific targets; thus, the potential mechanisms and pathways may differ [76]. Moreover, there is still a lack of clinical trials that currently deal with promoting healthy ageing via nutritional intervention [11,56].

## 4. Urolithin A and the CNS

### 4.1. Brain Health

For several years, increasing evidence of the neuroprotective properties of UA has been observed. In middle-aged and older adults with mild memory complaints, drinking 8 ounces of punicalagin-rich pomegranate juice for 4 weeks caused a significant improvement in verbal and visual memory that correlated well with plasma UA-glucuronide concentration [77]. Urine UA elevation was reported to also be associated with decreased age-related hippocamp atrophy—a biomarker of neurodegeneration and cognitive decline [78]. 

### 4.2. AD and Brain Injury

The above-mentioned favorable effects on the brain observed in humans with elevated UA levels in fluids make it a candidate preventive agent for neurodegenerative diseases. This idea is supported by in vivo findings that the administration of the compound improved cognition and reduced amyloid plaques, tau phosphorylation, neuronal loss, and neuroinflammation [14,15,16,17]. Mentioned effects are achievable due to the possibility of the free form of UA crossing the blood–brain barrier (BBB) in animal model studies [79,80]. However, there is no consensus as to whether the UA conjugates have the same properties or not [26].

The neuroprotective effect of UA administration was reported against neurodegeneration in a wide range of neuronal cell models, whereas the most considerable research was dedicated to AD and hypoxic-ischemic models. In AD model studies, the beneficial properties of UA were observed to be caused by the reduction of inflammation and improvement of mitochondrial health [15,16,55,69,81]. In mice and *C. elegans* AD models, supplementation with UA decreased the amyloid-β plaque burden in hippocampus areas and the cerebral cortices, which could be related to improved cognition [15,16,55,69,81]. The treatment also improved mitophagy and overall mitochondrial functions of the nerve cells [15,69]. UA administration was responsible for an increased microglial population, which contributed to the removal of plaques in APP/PS1 mice. In transgenic tau nematodes and mice, UA administration also decreased the phosphorylation of many p-tau sites, restoring memory impairments [15]. Moreover, inhibited cellular apoptosis in the cortex and hippocampal and partially restored hippocampal capacity for neurogenesis was reported in the UA-treated APP/PS1 mice [16]. Due to its anti-inflammatory properties, UA administration decreased levels of pro-inflammatory cytokines IL6 and TNF-α in AD mice [15,16,69]. Animal model studies support the UA’s protective role against amyloid-β peptide-induced toxicities and its contribution to the prevention of the onset of behavioral impairments [15,16,55,69,81] (Table 1 and Table 2).

Similarly, the treatment with UA was shown to have protective properties against brain injuries. UA treatment improved neurological deficit scores and reduced infarct volume or brain oedema in mice subjected to middle cerebral artery occlusion and moderate contusion injury [14,70,71]. UA rescued acute ischemic brain injury in mice subjected to middle cerebral artery occlusion and alleviated oxygen-glucose deprivation and reperfusion-induced injury both in N2a cells and primary cultured mice cortical neurons [14,71], and BBB permeability and neuronal apoptosis in the injured cortex [70]. Even though UA did not activate mitophagy, it reinforced autophagy in the injured cortex cells and led to suppressed ERS and related apoptosis in ischemic cells [14,70]. Moreover, the UA administration reduced neuroinflammation [70,71]. The neuroprotective effects were mediated by the inhibition of the PI3K/Akt/mTOR and Akt/IKK/NFκB signaling pathways [70], the activation of cerebral AMPK and IκBa, and the downregulation of Akt, P65NFκB, ERK, JNK, and P38MAPK [71] (Table 1 and Table 2).

### 4.3. Parkinson’s Disease

PD is a chronic and progressive neurodegenerative disorder characterized by a range of motor and non-motor symptoms [1]. The pathophysiology of the disease is thought to relate to α-synuclein aggregation, dysfunction of mitochondria, and neuroinflammation, which contribute to the process of neurodegeneration. A significant loss of dopaminergic neurons in the substantia nigra (SN) leads to a dopamine deficiency [2,82]. The developed imbalance between the direct and indirect pathways through the basal ganglia results in bradykinesia, slowness of movement and speed, one of Parkinsonism’s cardinal motor symptoms [83]. The etiology of PD remains multifactorial and complex, involving both genetic and environmental factors. The incidence and prevalence of the disease increase steadily with age [84]. 

Due to the increasing prevalence of the disease in an aging society, decreased life quality, and no available cure or treatment modifying its progress, there has been constant research for substances with neuroprotective properties [1,84]. In recent years, the research on the neuroprotective properties of UA has been increasing, mainly involving animal model studies on AD and brain injuries (Table 2), however only a few studies were performed using a PD model [72,73]. The findings of several experiments support the beneficial effects of UA on age-related disorders by increasing mitophagy, rescuing apoptosis, and attenuating inflammatory responses. It is only recently that researchers have begun to examine these mechanisms of the compound in the pathology of PD. In the study conducted on 1-methyl-4-phenyl-1,2,3,6-tetrahydropyridine (MPTP)- and 6-hydroxydopamine (6-OHDA)-induced PD mice models, UA administration resulted in neuroprotective effects. The treatment protected against motor deficits caused by both neurotoxins. It was accompanied by a decreased loss of dopaminergic neurons in the SN [72,73] and ameliorated neuroinflammation [72]. Further mechanistic study in LPS-induced BV2 microglial cells revealed favorable involvement of mitophagy, improvement in mitochondrial functions, and attenuation of the pro-inflammatory response upon UA exposition [72]. Maintaining mitochondrial homeostasis and the microglial mitochondria pool’s quality is believed to be a key factor in supporting brain health [72,85]. In both the MPTP-induced mice model and LPS-induced microglial model, UA downregulated NLR Family Pyrin Domain Containing 3 (NLRP3) inflammasome-mediated inflammation, alleviating the process of neuroinflammation [86]. The profusion of the specific multiprotein complex was observed in the microglia in the in vivo PD model [87]. NLRP3 inflammasome activation is known to be connected to the neuroinflammation process [85,86]. Accordingly, maintaining microglial mitophagy might contribute to the negative regulation of NLRP3 inflammasome-mediated inflammation [86,88]. A similar neuroprotective role of UA was also observed in 6-OHDA-induced neurotoxicity in PC12 cell cultures and mice models of PD [73]. The UA treatment prevented PC12 cells from 6-OHDA-induced cytotoxicity and apoptosis. Importantly, UA attenuated 6-OHDA-induced mitochondrial dysfunction and even improved mitochondrial biogenesis, which was demonstrated in PC12 cells and confirmed in the SN of mice challenged with 6-OHDA attributed to the increased expression of two mitochondrial proteins TOM20 and Tim23. Mechanistically, the biogenesis was stimulated via the SIRT1/PGC-1α signaling pathway [73] (Table 2). The NAD^+^-dependent deacetylase SIRT1 is a pro-survival protein inhibiting apoptosis and oxidative stress by regulating p53 and antioxidant defenses via FOXO family members, respectively. SIRT1 also interacts with PGC-1α to promote mitochondrial biogenesis and maintain mitochondrial homeostasis [89]. In the MPTP mouse model of PD, the activation of the SIRT1/PGC-1α pathway prevented dopaminergic neurons from oxidative stress and loss of neuronal viability [90]. Thus, the promotion of biogenesis via the SIRT1/PGC-1α signaling pathway could be a target for new therapies to prevent mitochondrial dysfunctions in neuronal injuries [89]. 

Moreover, given the evidence, which we previously summarized from human and experimental studies, that PD is a prion disorder originating in the gut [3], the ability of UA to modulate intestinal integrity and colonic immune milieu [10,38] may be considered for early dietary intervention to mitigate PD pathogenesis [91]. Recently, preliminary flow cytometric data by Ng and Andersen [91] showed that diet administration of UA significantly increased the anti-inflammatory colonic γδ T cells responsible for intestinal repair in aged mice with overexpressed human α-synuclein under the Thy1 promoter that was associated with improved cognitive behavior. The authors suggested the role of UA in the favorable retention of lymphocytes contributing to gut epithelial integrity and therefore involved in the targeted migration from the gut to the brain [91] (Table 2).

Another aspect of the role of UA in PD management is its inhibitory effects on monoamine oxidase (MAO). As the elevated MAO activity is responsible for the inactivation of monoamine neurotransmitters, such as dopamine, UA can contribute to alleviating the symptoms in PD patients [74].

Furthermore, UA could contribute to the overall beneficial impact reported for pomegranate consumption in PD models [26,79,92]. The reported neuroprotective effects of pomegranate could be explained since the fruit is a rich source of ellagitannins, natural precursors of UA [93,94,95]. The administration of pomegranate juice to rotenone-challenged rats improved postural stability, enhanced neuronal survival, and decreased dopamine depletion, oxidative damage, and α-synuclein aggregation [26,79]. Significantly, the UA concentration was 1.68 ± 0.25 ng/g in brain tissue and 18.75 ± 3.21 ng/mL in plasma [26]. Only recently, physiologically based pharmacokinetic modelling of the postbiotic supplement UA for predicting its bioavailability confirmed one-digit nanomolar concentrations in the brain [96]. The results contribute to the idea of UA crossing the BBB [79,80,97] and being the neurologically active metabolite of pomegranate juice [26,79]. As rotenone is a strong inhibitor of the mitochondrial complex I, the antioxidative effects of pomegranate treatment observed in rotenone-injected rats [26] can be explained by ameliorating mitochondrial dysfunction in oxidative stress mediated by UA [98]. Moreover, pomegranate treatment attenuated cylinder scores and catatonia rates dramatically declined in MPTP-induced PD mice models. Although the experiment improved movement and reduced levodopa-induced dyskinesia in the PD mice model, the authors did not associate them directly with the neuroprotective role of the UA administration [92]. In contrast, Tapias and co-workers reported that pomegranate juice failed to provide neuroprotection and even caused an exacerbation of rotenone-induced nigrostriatal degeneration in rats with an increased mortality rate. However, these adverse effects of pomegranate might have been ascribed to experimental protocol employing chronic administration of high doses of rotenone, causing severe oxidative stress and the death of the animals [99]. 

## 5. Summary and Future Perspectives

**Figure 3:**
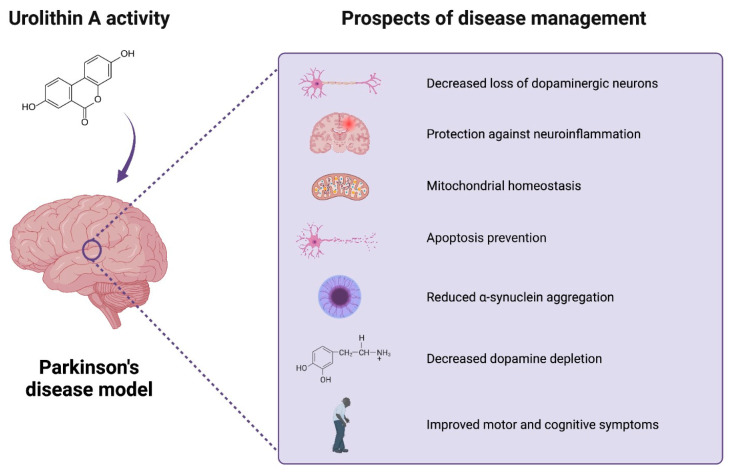
Urolithin A’s activity in Parkinson’s disease model studies. UA’s beneficial activity may include potential favorable effects of UA on brain health. The current research explores the potential neuroprotective effects of the compound in PD models. The beneficial role of the compound can be related to the reduction in neuroinflammation, loss of dopaminergic neurons, a-synuclein aggregation, and apoptosis, as well as improved mitochondrial, motor, and cognitive function. Created with BioRender.com.

A growing body of evidence suggests a variety of health benefits after UA administration. Numerous in vitro and in vivo experiments investigated the potential advantageous effects of the administration on ageing, muscle dysfunctions, cardiovascular disease, IBD, cancer, metabolic disorders, and brain health (Table 2). The neuroprotective properties in neurodegenerative disorders included improvement in cognition, motor activity, reduction of amyloid plaques, tau phosphorylation, neuronal loss, and neuroinflammation. In several PD models, UA treatment alleviated symptoms and provided protection against dopaminergic neurodegeneration. The role of the compound might be related to its ability to engage in mitochondrial homeostasis and induce mitophagy. The efficacy of the process tends to decrease with age, which is considered the risk factor for PD. Moreover, the metabolite provides anti-inflammatory properties (Figure 3).

Nevertheless, some study limitations should be acknowledged, such as shortages of disease models allowing only for an integrated view of a neuroprotective mechanism in terms of functional improvements with underlying changes in molecular signaling pathways, cell types, and brain networks. PD pathology is, however, diverse with multiple pathomechanisms involved; thus, no single model is currently able to recapitulate human disease [5]. Moreover, in animal studies, disease development occurs in a concise time span, and the duration of prevention or intervention is relatively short. Accordingly, the studies support a concept, but any medical implication is somewhat speculative [100].

Furthermore, to this day, there are numerous gaps in our knowledge of UA mechanisms of action, gut microbial ecologies, and specific impacts on health, including targeted organs and tissues, and primarily the effects on human organisms. Even though the compound can be produced from naturally occurring polyphenols in plant foods – ellagitannins and ellagic acid, the impact of direct UA supplementation on human health and its potential beneficial impact must be assessed. With regard to direct UA intake, the green synthetic access to new urolithin analogues [101] is a favorable circumstance, opening a new opportunity for UA-based neuroprotective strategy. However, despite observations of animal model studies on crossing the BBB and tissue distribution, there is still a lack of understanding of how those possibilities translate to clinical studies based on human participants. Safety, bioavailability, pattern of distribution, and mechanism of action in humans must be established. Regarding PD, given the recent advancement in detecting prodromal individuals before diagnosis and the potential prognostic value of the α-synuclein biomarker, research into selecting the right time for UA intervention shows promise to delay PD onset or progression to people at an increased risk [102]. Nevertheless, as predicted by computer modelling, low brain bioavailability of UA is orders of magnitudes lower than concentrations that provide neuroprotective effects [96], and thus should be managed by any supplementation scenario. Advanced nutritional approaches enabling the delivery of UA in a calibrated manner are likely to play a key role in bridging the gap created by the natural heterogeneity of the gut microbiome to deliver health benefits. In this context, recent advances in nanoplatforms for brain delivery of nutraceuticals can be considered [103,104].

Moreover, there is a need to identify the specific bacterial genera that can produce the metabolite to establish the specifics of urolithin-producing metabotypes and the related biochemical pathways and enzymes involved. In this context, UA was recently suggested as a potential biomarker of gut dysbiosis and disease stage in PD patients [105].

Taken together, the data gathered herein support the health-promoting activity of UA. Regarding PD, UA-based intervention offers new strategies to improve the prevention, treatment, and even diagnostics of the disease.

## Figures and Tables

**Table 1:** Biological activities of UA demonstrated in in vitro and in vivo model studies.

Dose and Route of Administration	Experimental Model	Outcomes	References
**Mitophagy and mitochondrial functions**			
50 µM	SH-SY5Y cells	↑ F-PINK-1, Parkin, Beclin-1, Bcl2L13, AMBRA1, and p-ULK1(Ser555)	[15]
200 mg/kg/d, i.g., for 2 months	6-month-old APP/PS1 mice	↑ relative neuronal mitophagy level ↑ mitophagy events ↓ damaged mitochondria ↑ F-PINK-1 in brain tissue	[15]
200 mg/kg/d, i.g., for 2 months	13-month-old 3 × TgAD mice	↑ relative neuronal mitophagy level ↑ mitophagy events in brain tissue	[15]
50 µM, **ad libitum**, for 10 days	*Caenorhabditis elegans*	↑ mRNA of the autophagy genes *Bec-1, Sqst-1 and Vps-34* ↑ mRNA of the mitophagy genes *Pink-1, Dct-1* ↑ mRNA of the mitophagy and biogenesis gene *Skn-1* in muscle tissue GFP–LGG-1-positive punctae	[29]
50 µM	C2C12 myoblasts Mode-K intestinal cells	↑ LC3-II/LC3-I, p-AMPKα ↑ SQSTM-1, Ub in mitochonfrial fraction ↑ autophagosomes ↑ autolysosomes ↓ mt DNA/nDNA ↑ mt chain subunits ↑ CII-driven respiration	[29]
50 mg/kg/d, p.o., for 34 weeks	16-month-old C57BL/6J mice	↑ LC3-II/LC3-I ↓ SQSTM-1 ↑ mRNA of autophagy genes *Becn1, Ulk1, Pik3c3, Atg8l, p62, Atg5, Atg7, Atg12, Lc3b, LAMP2* ↑ mRNA of mitophagy gene *Park2* ↑ p-AMPK ↑ Ub/SDHA, Ub/VDAC in muscle tissue	[29]
50 mg/kg/d, p.o., for 10 weeks	*Caenorhabditis elegans*/DMD model	↑ mRNA of mitophagy genes *Pink1, Pdr-1, Dct-1* ↑mRNA of autophagy genes *Bec-1, Vsp-34* ↑ mitochondrial network ↑ mitochondrial respiration ↑ citrate synthase activity ↑ mtDNA/nDNA in mucle tissue	[35]
50 mg/kg/d, p.o., for 10 weeks	13-week-old mdx mice/DMD model	↑ mRNA of mitophagy genes *Pink1, Park2, Park7, and Bnip3* ↑ mRNA of autophagy genes *Sqstm-1 and Becn1* ↑ p-S65-Ub, BNIP3, PARKIN, VDAC ↑ mt LC3-II in mucle tissue	[35]
25 µM	Primary myoblast cells derived from DMD patients	↑ mRNA of mitophagy genes *PINK1, PARK2, PARK7,* and *BNIP3* ↑ mRNA of autophagy genes *SQSTM-1, BECN1*	[35]
50 mg/kg/d, i.g., for 8 weeks	10-week-old C57BL/6 mice/STZ-induced model of type 2 diabetes	↑ LC3II/I, beclin1, ATG5, ↓ SQSTM-1 ↑ p-AKT, mTORC1 ↓ mitochondrial swelling in pancreatic tissue	[36]
**Anti-inflammatory activity**			
20 mg/kg, p.o., 10-day post-treatment	C57BL/6 mice/DSS-induced acute colitis	↓ IL-6, IL-1β, and TNF-α in serum ↓ MPO activity ↑ ZO-1, Ocln, Cldn4 ↓ F4/80^+^ CD11b^+^, CD4^+^ cells ↑ CD11c^+^, T-reg cells in the mesenteric lymph node	[10]
200 mg/kg/d, i.g., for 2 months	6-month-old APP/PS1 AD mice	↓ TNF-α, IL-6 ↑ IL-10 in brain tissue ↓ TNF-α, IL-6 ↑ IL-10 in microglia	[15]
300 mg/kg/d, p.o., for 14 days	28-week-old APP/PS1 AD mice	↓ mRNA of *Tnfα*, *Il6* and *Il1β* ↓ TNF-α, IL-6 and IL-1β ↓ IBA1 and GFAP in brain tissue	[16]
20 μg/d, i.p., for 12 weeks	C57BL/6 mice/HFD	↓ *Il1β* mRNA ↓ p-eIF2α, p-ERK ↑ IκBα LC3I/II in liver tissue ↓ M1 polarization (mRNA of *Cd11c*, *Tnfα, Il6, Il1β*, and *Mcp1*) ↑ M2 polarization (mRNA of *Ch3l3* and *Mgl2*) in peritoneal macrophages	[27]
50 mg/kg/d, i.g., for 8 weeks	10-week-old C57BL/6 mice/STZ-induced model of type 2 diabetes	↓ IL-1β, TNF-α ↑ IL-10 in plasma	[36]
100 mg/kg/d, i.p., for 5 days	C57BL/6 mice, cisplatin-induced nephrotoxicity model	↓ CD11b positive monocyte/macrophage ↓ mRNA of *Tnfα, Il23, Il18, Mip2* in kidney tissue	[37]
20 mg/kg/d, p.o., on 4th and 6th day of DSS cycle	C57BL/6 mice/DSS-, TNBS-induced colitis	↓ IL-6, IL-1β, and TNF-α in serum ↓ MPO activity ↓ Cldn4 in colon tissue	[38]
150 and 250 μM	Caco-2 and HT-29/B6/TNFα-induced barrier loss models	↑ TER ↓ claudin-2	[39]
25 mg/kg/d, p.o., 20 days	C57BL/6 mice/EAE model	↓ GFP+ cells in the brain and spinal cord ↓ M1-type microglia ↓ CD11c+ cells infiltrated into CNS ↓ CD45^high^ CD11b+, CD45^low^ CD11b+, CD11b+ MHCII+, CD11b+ TNF-α+, CD11b+ CD16/32+ cells	[40]
10, 40 μM, 2 h pretreatment	LPS-stimulated RAW264 macrophages	↓ TNF-α, IL-6, NO^−^, iNOS ↓ intracellular peroxides ↓ NADPH oxidase activity ↓ DNA binding activity of NF-κB and AP-1 ↓ NF-κB (p65) ↑ IκBα ↓ c-Jun, p-c-Jun, p-Akt, p-JNK, p-p38	[41]
**Antioxidant activity**			
20 μg/d, i.p., for 12 weeks	C57BL/6 mice/HFD	↑ mRNA of *Sod1* and *Sod2* in liver tissue	[27]
50 mg/kg/d, i.g., for 8 weeks	10-week-old C57BL/6 mice/STZ-induced model of type 2 diabetes	↑ GSH ↓ MDA in pancreas tissue	[36]
100 mg/kg/d, i.p., for 5 days	C57BL/6 mice, cisplatin-induced nephrotoxicity model	↑ CAT, GPx, SOD activity ↑ GSH ↓ GSSG, HNE, protein nitration, DNA fragmentation, *Nox2* mRNA in kidney tissue	[37]
0.5, 1, 2, 4 μM	N2a cells/H_2_O_2_	↑ CAT, SOD, GR, GPx activity ↓ ROS production, TBARS	[42]
**Apoptosis-modulating activity**			
300 mg/kg/d, i.g., for 14 days	28-week-old APP/PS1 AD mice	↑ NeuN^+^ cells ↓ TUNEL^+^ cells in brain tissue	[16]
100 mg/kg/d, i.p., for 5 days	C57BL/6 mice, cisplatin-induced nephrotoxicity model	↓ caspase 3 activity ↓ DNA fragmentation in kidney tissue	[37]
50 mg/kg, i.g., 3 times/week up to 19 days	C57BL/6J mice/cisplatin-induced AKI	↓ PARP1, TUNEL^+^ cells ↑ Bcl-2 in kidney tissue	[48]
10 μM, 6 h pretreatment	SH-SY5Y cells/H_2_O_2_	↓ apoptotic cells ↓ caspase-3, -9	[49]
1.25, 2.5, 5 μM, 6 h pretreatment	SK-N-MC cells/H_2_O_2_	↓ Bax/Bcl-2 ↓ cytochrome c, caspase-3, -9 ↓ PARP	[50]
25, 50, 100 μM	HT29, SW480, SW620 cells	↑ apoptotic cells ↑ ytochrome c, caspase-3, -9 ↑ p53, p21, XIAP ↓ Bcl-2 G2/M phase arrest	[51]
Treated with 40, 80 μM	LNCaP, 22RV1, PC3 cells	↑ p53, p21, MDM2 ↑ PUMA and NOXA	[52]
80 μM	HepG2.2.15 cells	↑ caspase-3 ↓ Bax/Bcl-2	[53]

↑ increase; ↓ decrease; AMBRA1, Autophagy and Beclin 1 Regulator; AP-1, Activator protein 1; APP/PS1, Amyloid precursor protein/presenilin 1; Atg12, Autophagy-related protein 12; Atg5, Autophagy-related protein 5; Atg7, Autophagy-related protein 7; Atg8l, Autophagy-related protein 8l; Bax, Bcl-2 associated X protein; Bcl-2, B-cell lymphoma 2; Bcl2L13, Bcl2 like protein13; Bec-1, ortholog of human BECN1/Beclin 1; Becn1, Beclin-1 gene; Bnip3, Bcl-2 interacting protein 3; c-Jun, transcription factor Jun; CAT, Catalase; CD11b, Integrin alpha M; CD11c, Integrin alpha X; CD16/32, IgG Fc receptor III (FcR III)/IgG Fc receptor II FcR II; CD4, CD4 T lymphocytes; CD45, Leucocyte common antigen; Ch3l3, Chitinase-like 3 protein; CII, Complex II; Cldn4, Claudin 4; CNS, Central nervous system; Dct-1, Dopachrome Tautomerase; DMD, Duchenne muscular dystrophy; DSS, Dextran sulfate sodium; EAE, Experimental autoimmune encephalomyelitis; F4/80, Membrane glycoprotein; F-PINK-1, Full length PTEN-induced kinase 1; GFAP, Glial fibrillary acidic protein; GFP–LGG-1, Green fluorescent protein–*C. elegans* Microtubule-associated proteins 1A/1B light chain 3B (LC3); GPx, Glutathione peroxidase; GR, Glutathione reductase; GSH, Reduced glutathione; GSSG, Oxidized glutathione; HFD, High fat diet; HNE, 4-Hydroxynonenal; IBA1, Ionized calcium-binding adaptor molecule 1; IL-1β, Interleukin-1β; IL-10, Interleukin-10; IL-18, Interleukin-18; IL-23, Interleukin-23; IL-6, Interleukin-6; iNOS, Inducible nitric oxide synthase; IκBα, Nuclear factor-κB inhibitor alpha; LAMP2, Lysosomal Associated Membrane Protein 2; LC3-I, Cytosolic form of microtubule-associated protein 1A/1B-light chain 3; LC3-II, Membrane-bound form of microtubule-associated protein 1A/1B-light chain 3; Lc3b, Microtubule-associated proteins 1A/1B light chain 3B; LPS, Lipopolysaccharides; M1, Type of classically activated macrophages; M2, Type of alternatively activated macrophages; Mcp1, Monocyte chemoattractant protein-1; MDA, Malondialdehyde; MDM2, E3 ubiquitin-protein ligase that mediates ubiquitination of p53/TP53; Mgl2, Macrophage galactose N-acetyl-galactosamine specific lectin 2; MHCII, The major histocompatibility complex class II; MIP2, Macrophage inflammatory protein-2; MPO, Myeloperoxidase; mTORC1, Mammalian target of rapamycin complex 1; mt-, mitochondrial; N2a, neuro-2a cells; NADPH, Nicotinamide adenine dinucleotide phosphate; NeuN, Neuron-specific nuclear protein; NF-κB, Nuclear factor kappa-B signaling pathway; NO-, Nitric oxide anion; NOX2, NADPH oxidase 2; NOXA, Transcriptional target of p53; Ocln, Occludin; p-AKT, Phosphorylated protein kinase B; p-AMPK, Phosphorylated AMP-activated protein kinase; p-AMPKα, Phosphorylated AMP-activated protein kinase alpha; p-c-Jun, Phosphorylated transcription factor Jun; p-eIF2α, Phosphorylated eukaryotic translation initiation factor 2A; p-ERK, Phosphorylated extracellular signal-regulated kinase; p-JNK, Phosphorylated c-Jun N-terminal kinase; p-p38, Phosphorylated mitogen-activated protein kinase; p-S65-Ub, PINK1-phosphorylated ubiquitin; p-ULK1(Ser555), Phosphorylated at Ser555 serine/threonine-specific protein kinase; p21, cyclin-dependent kinase inhibitor; p53, Tumor suppressor critical for apoptosis; p62, Autophagic adaptor; p65, nuclear factor NF-kappa-B p65 subunit; Park2, Parkin RBR E3 ubiquitin protein ligase; Park7, Parkinsonism associated deglycase; Parkin, 465-amino acid residue E3 ubiquitin ligase protein; PARP, Poly (ADP-ribose) polymerase; PARP1, Poly (ADP-ribose) polymerase 1; Pdr-1, E3 ubiquitin-protein ligase parkin; Pik3c3, Phosphatidylinositol 3-kinase catalytic subunit type 3; Pink-1, PTEN-induced kinase 1; PUMA, p53 upregulated modulator of apoptosis; ROS, Reactive oxygen species; SDHA, Succinate Dehydrogenase Complex Flavoprotein Subunit A; Skn-1, Negative Regulator of DAF-16; SOD, Superoxide dismutase; SOD1, Superoxide dismutase type 1; SOD2, Superoxide dismutase type 2; SQSTM-1, Sequestosome 1; STZ, Streptozotocin; TBARS, Thiobarbituric acid reactive substances; TER, Transepithelial resistance; TNBS, 2,4,6-trinitrobenzenesulfonic acid; TNF-α, Tumor necrosis factor alpha; TUNEL, Terminal deoxynucleotidyl transferase dUTP Nick-End Labeling; Ub, Ubiquitin; Ulk1, Unc-51 Like Autophagy Activating Kinase 1; VDAC, Voltage Dependent Anion Channel 1; Vps-34, Phosphatidylinositol 3-kinase; XIAP, X-linked inhibitor of apoptosis; ZO-1, Zonula occludens-1.

**Table 2:** Biological effects of UA in different health conditions and diseases.

Dose and Route of Administration	Experimental Model	Outcomes	References
**Longevity**			
500 mg or 1000 mg/d, p.o. for 4 months	Middle-aged adults between 40 to 65 years old	↑muscle strength, aerobic-endurance, physical performance ↓ acylcarnitines ↓ CRP, IL-1β, TNF-α, IFNγ in plasma	[11]
50 µM, **ad libitum**, for 10 days	*Caenorhabditis elegans*	↑ lifespan, mobility, ↑ pharyngeal pumping ↑ mitochondrial respiratory capacity ↓ dysfunctional mitochondria in muscle tissue	[29]
50 mg/kg/d, p.o., for 10 weeks	13-week-old mdx mice DMD model	↑ improved muscle morphology ↑ motility ↓ muscle fiber degeneration ↑ eMyHC, dystrophin in muscle tissue	[35]
10 mg/kg/d, p.o., for 16 weeks	C57BL/6 mice	↑muscle strength, mobility, and exercise performance	[54]
1000 mg/d, p.o., for 4 months	Adults aged 65 to 90 years	↑ muscle endurance, physical performance ↓ acylcarnitines, ceramides ↓ CRP in plasma	[56]
5 mg/kg/d, p.o., 10 months in alternate weeks (1 week on, 1 week off)	Female B6129SF2/J and male C57BL/6NJ/aging model	↑ lifespan	[55]
**Cardiovascular health**			
50 mg/kg/d, p.o., for 20 weeks	C57BL/6 mice/HFD	↑ PINK1/Parkin-dependent mitophagy ↓ mitochondrial defects ↑ cardiac diastolic function	[57]
3 mg/kg/d, p.o., for 3 weeks	Wistar rats/HFD + ballon injury in the aorta	↓ TC, TG and LDL ↓ Ang II ↓ aortic edema	[58]
1 mg/kg, i.p., 24 h and 1 h pretreatment	C57BL/6 mice/H/R	↓ myocardial infarct sizes ↓ TUNEL^+^ cells ↑ ejection fraction, fractional shortening ↓ CK, LDH in serum	[59]
10 mg/d for 12 weeks	Healthy volunteers 40–65 years old, UA non-producer or low producer	↑ FMD score	[60]
**Metabolic dysfunctions**			
20 μg/d, i.p., for 12 weeks	C57BL/6 mice/HFD	↓ hepatic TG ↓ IR, adipocyte hypertrophy ↓ macrophage infiltration into the adipose tissue ↓ M1/M2	[27]
50 mg/kg/d, i.g., for 8 weeks	10-week-old C57BL/6 mice/STZ-induced model of type 2 diabetes	↓ BW ↓ FBG, GHb ↓ pancreatic histopathological damages ↑ HOMA-β	[36]
30 mg/kg/d, i.g., for 10 weeks	C57BL/6 mice/HFD	↓ BW, fat mass ↓ plasma glucose, insulin ↑ thermogenesis in brown adipose tissue ↑ browning of white adipose tissue	[61]
13 mg/kg/d, p.o., for 8 weeks	DBA2J mice/HFD/HSD	↓ FBG ↓ serum TG, FFA, adiponectin ↓ IR	[62]
**Cancer**			
25–100 μM	PC-3 cells, C4-2B cells	↓ cell proliferation ↑ apoptosis	[63]
50 mg/kg, p.o.; 5 days/week, for 4–5 weeks	C4-2B xenografted mice PC-3 xenografted mice	↓ tumor volume ↓ Ki67, AR, and pAKT in tumor samples	[63]
50–200 μM	HepG2 cells	↓ Cell survival ↓ Wnt/β-catenin signaling; β-catenin ↓ c-Myc, cyclin D1, p-c-Jun ↑ TP53, BAX, PUMA, NOXA, p53, p-p53 ↑ Caspase-3, p-p38	[64]
Pretreatment with 5–25 μM	A549, H460, H1299 cells	↓ EMT ↓ Snail expression	[65]
0.5–10 μM	HCT-116, Caco-2, and HT-29 cells	↓ Colony formation G2/M arrest (Caco-2, HCT-116) ↑ Senescence-associated β–galactosidase activity (HCT-116) ↑ p53 and p21Cip1/Waf1 expression (HCT-116)	[66]
30 μM	HCT116 cells	↓ Cell growth ↑ p53, p21, TIGAR expression	[67]
1.5 μM	SW620 cells	↓ Proliferation, MMP-9 activity ↑ Autophagy, LC3 G2/M arrest ↑ Apoptosis, necrosis	[68]
**Alzheimer’s disease**			
200 mg/kg/d, i.g., for 2 months	6-month-old APP/PS1 AD mice	↑ Cognitive behavior ↓ Aβ 1–40 and 1–42	[15]
300 mg/kg/d, p.o., for 14 days	28-week-old APP/PS1 AD mice	↓ learning and memory deficits ↓ Aβ 42 in the cerebral cortex and hippocampus	[16]
5 mg/kg/d, p.o., 10 months in alternate weeks (1 week on, 1 week off)	3xTg-AD mice	↓ Aβ 42 in hippocampus ↑ learning and exploratory behavior	[55]
30 μM	HT22 cells/Aβ oligomers	↓ Aβ, SQSTM1, LC3	[55]
Intraperitoneal injection, 2.5 mg/kg, 3 times per week for 4 months	7-month-old hAbKI mice	↑ Cognitive behavior ↓ Aβ 1–40 and 1–42 ↓ mitochondria fission proteins Drp1, Fis1 ↑ mitochondria fusion proteins Mfn2, Opa1 ↑ mitochondrial biogenesis proteins PGC1α, Nrf1, Nrf2, TFAM ↑ mitophagy proteins PINK1, Parkin ↑ synaptic proteins synaptophysin PSD95 ↑ autophagy proteins ATG5, Beclin, BCL2, LC3B-I, LC3B-II ↓ microglia IBA-1, astrocytes GFAP, neuronal NeuN in brain tissue	[69]
**Brain injury**			
2.5, 5 mg/kg, i.p., twice s1 h and 24 h before MCAO surgery	C57BL/6 mice/MCAO	↓ infarct volume, NDS ↓ mRNA of autophagy genes *Atf6* and *Chop* in the brain tissue	[14]
30 μM	N2a cells, primary cultured neurons/OGD/R injury	↑ cell viability ↓LDH ↑ LC3II, ↓p62 ↓ mRNA of autophagy genes *Atf6* and *Chop*	[14]
2.5 mg/kg, i.p., immediately after controlled injury and every 24 h for 3 days	C57BL/6J/CTI	↓ NSS score, brain edema ↓ TUNEL^+^/NeuN^+^ cells ↓ caspase-3 ↑ bcl-2 ↑ LC3-II/LC3-I ↓ p62 ↓ p-Akt/Akt, p-mTOR/mTOR, p-IKKα/IKKα, p-NFκB/NFκB in the hippocampus	[70]
1.5, 2 mg/kg, i.p., twice 1 h and 24 h before MCAO surgery	Mice/MCAO	↓ infarct volume, NDS in hippocampus ↓ spatial memory deficits ↑ Nissl^+^ cells ↓ TUNEL^+^ cells ↓ Bax, caspase-3, ↑ Bcl-2 in the hippocampus	[71]
**Parkinson’s disease**			
20 mg/kg/d, i.p., 7-day pretreatment	Mice/MPTP	↑ Nissl^+^, TH^+^neurons ↓ motor deficits ↓ p62 ↑ LC3II/I ↓ NLRP3, caspase-1, IL-1β ↓ IBA1^+^, GFAP^+^ cells in the SN ↓ NLRP3 inflammasome activation ↓ neuroinflammation	[72]
10 μM	BV2 cells/LPS	↓ p62 ↑ LC3II/I ↑ PINK1 and Parkin ↓ Tim23 and Tom20 ↓ mRNA of *Il1β, Tnfα, iNos*, and *Cox2* ↓ NO, NLRP3, caspase-1, IL-1β ↑ MMP, mitochondrial metabolism	[72]
10 μM	PC12 cells/6-OHDA	↑ cell viability ↓ apoptotic rate ↑ MMP, ↑ Tim23, Tom20 ↑ TFAM, PGC1α, SIRT1	[73]
10 mg/kg/d, i.p., 7-day pretreatment	C57BL/6J mice/6-OHDA	↑ Nissl^+^, TH^+^neurons ↓ motor deficits ↑ TFAM, PGC1α, SIRT1 In the SN	[73]
Diet exposition (exposure details not available)	9-month-old Thy-1α-syn mice	↑ Blood colonic γδ T cells ↑ novel object recognition	[74]

↑ increase; ↓ decrease; 6-OHDA, 6-hydroxydopamine; Ang II, Angiotensin II; AR, Androgen receptor; APP/PS1, Amyloid precursor protein/presenilin 1; ATF6, Activating transcription factor 6; Atg5, Autophagy-related protein 5; Aβ, Amyloid beta; Bax, Bcl-2 associated X protein; Bcl-2, B-cell lymphoma 2; BV-2 cells, microglial cell derived from C57/BL6 murine; BW, Body weight; c-Myc, proto-oncogene Myc; CHOP, CCAAT-enhancer-binding homologous protein; CK, Creatine kinase; COX-2, Prostaglandin-endoperoxide synthase 2; CRP, C-reactive protein; CTI, Controlled Cortical Impact; DMD, Duchenne muscular dystrophy; Drp1, Dynamin-related protein 1; EMT, Epithelial-to-mesenchymal transition; eMyHC, Embryonic myosin heavy chain expression; FBG, Fasting-blood-glucose; FFA, Free fatty acids; Fis1, Fission 1; FMD, Flow-mediated vasodilatation; GFAP, Glial fibrillary acidic protein; GHb, Glycated hemoglobin; H/R, Hypoxia/reoxygenation; hAbKI, Humanized homozygous amyloid beta knockin; HFD, High-fat diet; HOMA-β, Homeostasis model assessment-β; HSD, High Sugar Diet; IBA1, Ionized calcium-binding adaptor molecule 1; IL-1β, Interleukin-1β; IKKα, IκB kinase alpha; INFγ, Interferon gamma; iNOS, Inducible nitric oxide synthase; IR, Insulin resistance; Ki67, Antigen Ki-67; Lc3, Microtubule-associated proteins 1A/1B light chain 3B; LC3B-I, Microtubule-associated protein 1A/1B-light chain 3B 1; LC3B-II, Microtubule-associated protein 1A/1B-light chain 3B 2; LDH, Lactate dehydrogenase; LDL, Low-density lipoprotein; LPS, Lipopolysaccharides; M1, Type of classically activated macrophages; M2, Type of alternatively activated macrophages; MCAO, Middle cerebral artery occlusion; Mfn2, Mitochondrial fusion 2; MMP, Mitochondrial membrane potential; MMP-9, Matrix metalloproteinase-9; MPTP, 1-methyl-4-phenyl-1,2,3,6-tetrahydropyridine; mTOR, Mammalian target of rapamycin; NDS, Neurological deficit scores; NeuN, Neuron-specific nuclear protein; NF-κB, Nuclear factor kappa-B signaling pathway; NLRP3, NLR Family Pyrin Domain Containing 3; NO, Nitric oxide; NOXA, BH3-only BCL2 family protein; Nrf1, Nuclear transcription factor 1; Nrf2, Nuclear transcription factor 2; NSS, Neurological Severity Score; OGD/R, Oxygen-glucose deprivation/reoxygenation; Opa1, Optic atrophy 1; p-AKT, Phosphorylated protein kinase B; p-c-Jun, Phosphorylated transcription factor Jun; p-IKKα, Phosphorylated IκB kinase alpha; p-mTOR, Phosphorylated mammalian target of rapamycin; p-NF-κB, Phosphorylated Nuclear factor kappa-B signaling pathway; p-p38, Phosphorylated mitogen-activated protein kinase; p-p53, Phosphorylated Tumor suppressor critical for apoptosis; p21, cyclin-dependent kinase inhibitor; p53, Tumor suppressor critical for apoptosis; p62, Autophagic adaptor; p21Cip1/Waf1, Cyclin-dependent kinase inhibitor regulated by the tumor suppressor p53; Parkin, 465-amino acid residue E3 ubiquitin ligase protein; PC12 cells, cell line derived from a pheochromocytoma of the rat adrenal medulla; PGC1α, Peroxisome proliferation-activated receptor gamma coactivator 1-alpha; PINK1, PTEN-induced kinase 1; PSD95, Postsynaptic density protein 95; PUMA, p53 upregulated modulator of apoptosis; SIRT1, Sirtuin 1; N2a, neuro-2a cells; SN, Substantia nigra; SQSTM-1, Sequestosome 1; STZ, Streptozotocin; TC, Total cholesterol; TFAM, Human mitochondrial transcription factor A; TG, Triglycerides; TH, Tyrosine hydroxylase; Thy1-αSyn mouse overexpressing human α-synuclein under the Thy1 promoter; TIGAR, Tumor protein P53-induced glycolysis and apoptosis regulator; Tim23, Mitochondrial import inner membrane translocase subunit TIM23; Tom20, Translocase of outer mitochondrial membrane 20; TP53, Gene encoding protein p53; TNF-α, Tumor necrosis factor alpha; TUNEL, Terminal deoxynucleotidyl transferase dUTP Nick-End Labeling; Wnt, Wingless and Int signaling pathway.

## References

[1] Kalia L.V., Lang A.E. (2015). Parkinson’s disease. Lancet.

[2] Chen Z., Li G., Liu J. (2020). Autonomic dysfunction in Parkinson’s disease: Implications for pathophysiology, diagnosis, and treatment. Neurobiol. Dis..

[3] Kujawska M., Jodynis-Liebert J. (2018). What is the Evidence That Parkinson’s Disease is a Prion Disorder, Which Originates in the Gut?. Int. J. Mol. Sci..

[4] Armstrong M.J., Okun M.S. (2020). Diagnosis and Treatment of Parkinson Disease: A Review. JAMA.

[5] Kujawska M., Jodynis-Liebert J. (2018). Polyphenols in Parkinson’s Disease: A Systematic Review of In Vivo Studies. Nutrients.

[6] Gupta A., Singh A.K., Kumar R., Jamieson S., Pandey A.K., Bishayee A. (2021). Neuroprotective Potential of Ellagic Acid: A Critical Review. Adv. Nutr..

[7] Garcia-Villalba R., Gimenez-Bastida J.A., Cortes-Martin A., Avila-Galvez M.A., Tomas-Barberan F.A., Selma M.V., Espin J.C., Gonzalez-Sarrias A. (2022). Urolithins: A Comprehensive Update on their Metabolism, Bioactivity, and Associated Gut Microbiota. Mol. Nutr. Food Res..

[8] D’Amico D., Andreux P.A., Valdes P., Singh A., Rinsch C., Auwerx J. (2021). Impact of the Natural Compound Urolithin A on Health, Disease, and Aging. Trends Mol. Med..

[9] Denk D., Petrocelli V., Conche C., Drachsler M., Ziegler P.K., Braun A., Kress A., Nicolas A.M., Mohs K., Becker C. (2022). Expansion of T memory stem cells with superior anti-tumor immunity by Urolithin A-induced mitophagy. Immunity.

[10] Ghosh S., Moorthy B., Haribabu B., Jala V.R. (2022). Cytochrome P450 1A1 is essential for the microbial metabolite, Urolithin A-mediated protection against colitis. Front. Immunol..

[11] Singh A., D’Amico D., Andreux P.A., Fouassier A.M., Blanco-Bose W., Evans M., Aebischer P., Auwerx J., Rinsch C. (2022). Urolithin A improves muscle strength, exercise performance, and biomarkers of mitochondrial health in a randomized trial in middle-aged adults. Cell Rep. Med..

[12] Djedjibegovic J., Marjanovic A., Panieri E., Saso L. (2020). Ellagic Acid-Derived Urolithins as Modulators of Oxidative Stress. Oxid. Med. Cell Longev..

[13] Kujawska M., Jodynis-Liebert J. (2020). Potential of the ellagic acid-derived gut microbiota metabolite—Urolithin A in gastrointestinal protection. World J. Gastroenterol..

[14] Ahsan A., Zheng Y.R., Wu X.L., Tang W.D., Liu M.R., Ma S.J., Jiang L., Hu W.W., Zhang X.N., Chen Z. (2019). Urolithin A-activated autophagy but not mitophagy protects against ischemic neuronal injury by inhibiting ER stress in vitro and in vivo. CNS Neurosci. Ther..

[15] Fang E.F., Hou Y., Palikaras K., Adriaanse B.A., Kerr J.S., Yang B., Lautrup S., Hasan-Olive M.M., Caponio D., Dan X. (2019). Mitophagy inhibits amyloid-beta and tau pathology and reverses cognitive deficits in models of Alzheimer’s disease. Nat. Neurosci..

[16] Gong Z., Huang J., Xu B., Ou Z., Zhang L., Lin X., Ye X., Kong X., Long D., Sun X. (2019). Urolithin A attenuates memory impairment and neuroinflammation in APP/PS1 mice. J. Neuroinflammation.

[17] Busto R., Serna J., Perianes-Cachero A., Quintana-Portillo R., Garcia-Seisdedos D., Canfran-Duque A., Paino C.L., Lerma M., Casado M.E., Martin-Hidalgo A. (2018). Ellagic acid protects from myelin-associated sphingolipid loss in experimental autoimmune encephalomyelitis. Biochim. Biophys. Acta Mol. Cell Biol. Lipids.

[18] Senobari Z., Karimi G., Jamialahmadi K. (2022). Ellagitannins, promising pharmacological agents for the treatment of cancer stem cells. Phytother. Res..

[19] Zary-Sikorska E., Fotschki B., Jurgonski A., Kosmala M., Milala J., Kolodziejczyk K., Majewski M., Ognik K., Juskiewicz J. (2020). Protective Effects of a Strawberry Ellagitannin-Rich Extract against Pro-Oxidative and Pro-Inflammatory Dysfunctions Induced by a High-Fat Diet in a Rat Model. Molecules.

[20] Tomás-Barberán F.A., González-Sarrías A., García-Villalba R., Núñez-Sánchez M.A., Selma M.V., García-Conesa M.T., Espín J.C. (2017). Urolithins, the rescue of “old” metabolites to understand a “new” concept: Metabotypes as a nexus among phenolic metabolism, microbiota dysbiosis, and host health status. Mol. Nutr. Food Res..

[21] Garcia-Villalba R., Tomas-Barberan F.A., Iglesias-Aguirre C.E., Gimenez-Bastida J.A., Gonzalez-Sarrias A., Selma M.V., Espin J.C. (2023). Ellagitannins, urolithins, and neuroprotection: Human evidence and the possible link to the gut microbiota. Mol. Aspects Med..

[22] Garcia-Villalba R., Selma M.V., Espin J.C., Tomas-Barberan F.A. (2019). Identification of Novel Urolithin Metabolites in Human Feces and Urine after the Intake of a Pomegranate Extract. J. Agric. Food Chem..

[23] Bobowska A., Granica S., Filipek A., Melzig M.F., Moeslinger T., Zentek J., Kruk A., Piwowarski J.P. (2021). Comparative studies of urolithins and their phase II metabolites on macrophage and neutrophil functions. Eur. J. Nutr..

[24] Cortes-Martin A., Garcia-Villalba R., Garcia-Mantrana I., Rodriguez-Varela A., Romo-Vaquero M., Collado M.C., Tomas-Barberan F.A., Espin J.C., Selma M.V. (2020). Urolithins in Human Breast Milk after Walnut Intake and Kinetics of Gordonibacter Colonization in Newly Born: The Role of Mothers’ Urolithin Metabotypes. J. Agric. Food Chem..

[25] Gu J., Thomas-Ahner J.M., Riedl K.M., Bailey M.T., Vodovotz Y., Schwartz S.J., Clinton S.K. (2019). Dietary Black Raspberries Impact the Colonic Microbiome and Phytochemical Metabolites in Mice. Mol. Nutr. Food Res..

[26] Kujawska M., Jourdes M., Kurpik M., Szulc M., Szaefer H., Chmielarz P., Kreiner G., Krajka-Kuzniak V., Mikolajczak P.L., Teissedre P.L. (2019). Neuroprotective Effects of Pomegranate Juice against Parkinson’s Disease and Presence of Ellagitannins-Derived Metabolite-Urolithin A-In the Brain. Int. J. Mol. Sci..

[27] Toney A.M., Fan R., Xian Y., Chaidez V., Ramer-Tait A.E., Chung S. (2019). Urolithin A, a Gut Metabolite, Improves Insulin Sensitivity Through Augmentation of Mitochondrial Function and Biogenesis. Obesity (Silver Spring).

[28] Heilman J., Andreux P., Tran N., Rinsch C., Blanco-Bose W. (2017). Safety assessment of Urolithin A, a metabolite produced by the human gut microbiota upon dietary intake of plant derived ellagitannins and ellagic acid. Food Chem. Toxicol..

[29] Ryu D., Mouchiroud L., Andreux P.A., Katsyuba E., Moullan N., Nicolet-Dit-Félix A.A., Williams E.G., Jha P., Lo Sasso G., Huzard D. (2016). Urolithin A induces mitophagy and prolongs lifespan in C. elegans and increases muscle function in rodents. Nat. Med..

[30] Cortes-Martin A., Garcia-Villalba R., Gonzalez-Sarrias A., Romo-Vaquero M., Loria-Kohen V., Ramirez-de-Molina A., Tomas-Barberan F.A., Selma M.V., Espin J.C. (2018). The gut microbiota urolithin metabotypes revisited: The human metabolism of ellagic acid is mainly determined by aging. Food Funct..

[31] Xian W., Yang S., Deng Y., Yang Y., Chen C., Li W., Yang R. (2021). Distribution of Urolithins Metabotypes in Healthy Chinese Youth: Difference in Gut Microbiota and Predicted Metabolic Pathways. J. Agric. Food Chem..

[32] Singh A., D’Amico D., Andreux P.A., Dunngalvin G., Kern T., Blanco-Bose W., Auwerx J., Aebischer P., Rinsch C. (2022). Direct supplementation with Urolithin A overcomes limitations of dietary exposure and gut microbiome variability in healthy adults to achieve consistent levels across the population. Eur. J. Clin. Nutr..

[33] Palikaras K., Lionaki E., Tavernarakis N. (2018). Mechanisms of mitophagy in cellular homeostasis, physiology and pathology. Nat. Cell Biol..

[34] Andreux P.A., Blanco-Bose W., Ryu D., Burdet F., Ibberson M., Aebischer P., Auwerx J., Singh A., Rinsch C. (2019). The mitophagy activator urolithin A is safe and induces a molecular signature of improved mitochondrial and cellular health in humans. Nat. Metab..

[35] Luan P., D’Amico D., Andreux P.A., Laurila P.P., Wohlwend M., Li H., Imamura de Lima T., Place N., Rinsch C., Zanou N. (2021). Urolithin A improves muscle function by inducing mitophagy in muscular dystrophy. Sci. Transl. Med..

[36] Tuohetaerbaike B., Zhang Y., Tian Y., Zhang N.N., Kang J., Mao X., Zhang Y., Li X. (2020). Pancreas protective effects of Urolithin A on type 2 diabetic mice induced by high fat and streptozotocin via regulating autophagy and AKT/mTOR signaling pathway. J. Ethnopharmacol..

[37] Jing T., Liao J., Shen K., Chen X., Xu Z., Tian W., Wang Y., Jin B., Pan H. (2019). Protective effect of urolithin a on cisplatin-induced nephrotoxicity in mice via modulation of inflammation and oxidative stress. Food Chem. Toxicol..

[38] Singh R., Chandrashekharappa S., Bodduluri S.R., Baby B.V., Hegde B., Kotla N.G., Hiwale A.A., Saiyed T., Patel P., Vijay-Kumar M. (2019). Enhancement of the gut barrier integrity by a microbial metabolite through the Nrf2 pathway. Nat. Commun..

[39] Hering N.A., Luettig J., Jebautzke B., Schulzke J.D., Rosenthal R. (2021). The Punicalagin Metabolites Ellagic Acid and Urolithin A Exert Different Strengthening and Anti-Inflammatory Effects on Tight Junction-Mediated Intestinal Barrier Function In Vitro. Front. Pharmacol..

[40] Shen P.X., Li X., Deng S.Y., Zhao L., Zhang Y.Y., Deng X., Han B., Yu J., Li Y., Wang Z.Z. (2021). Urolithin A ameliorates experimental autoimmune encephalomyelitis by targeting aryl hydrocarbon receptor. EBioMedicine.

[41] Komatsu W., Kishi H., Yagasaki K., Ohhira S. (2018). Urolithin A attenuates pro-inflammatory mediator production by suppressing PI3-K/Akt/NF-kappaB and JNK/AP-1 signaling pathways in lipopolysaccharide-stimulated RAW264 macrophages: Possible involvement of NADPH oxidase-derived reactive oxygen species. Eur. J. Pharmacol..

[42] Casedas G., Les F., Choya-Foces C., Hugo M., Lopez V. (2020). The Metabolite Urolithin-A Ameliorates Oxidative Stress in Neuro-2a Cells, Becoming a Potential Neuroprotective Agent. Antioxidants.

[43] Liu W., Ma H., Frost L., Yuan T., Dain J.A., Seeram N.P. (2014). Pomegranate phenolics inhibit formation of advanced glycation endproducts by scavenging reactive carbonyl species. Food Funct..

[44] Verzelloni E., Pellacani C., Tagliazucchi D., Tagliaferri S., Calani L., Costa L.G., Brighenti F., Borges G., Crozier A., Conte A. (2011). Antiglycative and neuroprotective activity of colon-derived polyphenol catabolites. Mol. Nutr. Food Res..

[45] Di Stasi L.C. (2023). Natural Coumarin Derivatives Activating Nrf2 Signaling Pathway as Lead Compounds for the Design and Synthesis of Intestinal Anti-Inflammatory Drugs. Pharmaceuticals.

[46] Lou L., Wang M., He J., Yang S., Meng F., Wang S., Jin X., Cai J., Cai C. (2023). Urolithin A (UA) attenuates ferroptosis in LPS-induced acute lung injury in mice by upregulating Keap1-Nrf2/HO-1 signaling pathway. Front. Pharmacol..

[47] Mazumder M.K., Choudhury S., Borah A. (2019). An in silico investigation on the inhibitory potential of the constituents of Pomegranate juice on antioxidant defense mechanism: Relevance to neurodegenerative diseases. IBRO Rep..

[48] Zou D., Ganugula R., Arora M., Nabity M.B., Sheikh-Hamad D., Kumar M. (2019). Oral delivery of nanoparticle urolithin A normalizes cellular stress and improves survival in mouse model of cisplatin-induced AKI. Am. J. Physiol. Renal Physiol..

[49] Gonzalez-Sarrias A., Nunez-Sanchez M.A., Tomas-Barberan F.A., Espin J.C. (2017). Neuroprotective Effects of Bioavailable Polyphenol-Derived Metabolites against Oxidative Stress-Induced Cytotoxicity in Human Neuroblastoma SH-SY5Y Cells. J. Agric. Food Chem..

[50] Kim K.B., Lee S., Kim J.H. (2020). Neuroprotective effects of urolithin A on H(2)O(2)-induced oxidative stress-mediated apoptosis in SK-N-MC cells. Nutr. Res. Pract..

[51] El-Wetidy M.S., Ahmad R., Rady I., Helal H., Rady M.I., Vaali-Mohammed M.A., Al-Khayal K., Traiki T.B., Abdulla M.H. (2021). Urolithin A induces cell cycle arrest and apoptosis by inhibiting Bcl-2, increasing p53-p21 proteins and reactive oxygen species production in colorectal cancer cells. Cell Stress. Chaperones.

[52] Mohammed Saleem Y.I., Albassam H., Selim M. (2020). Urolithin A induces prostate cancer cell death in p53-dependent and in p53-independent manner. Eur. J. Nutr..

[53] Qiu Z., Zhou J., Zhang C., Cheng Y., Hu J., Zheng G. (2018). Antiproliferative effect of urolithin A, the ellagic acid-derived colonic metabolite, on hepatocellular carcinoma HepG2.2.15 cells by targeting Lin28a/let-7a axis. Braz. J. Med. Biol. Res..

[54] Ghosh N., Das A., Biswas N., Gnyawali S., Singh K., Gorain M., Polcyn C., Khanna S., Roy S., Sen C.K. (2020). Urolithin A augments angiogenic pathways in skeletal muscle by bolstering NAD(+) and SIRT1. Sci. Rep..

[55] Ballesteros-Alvarez J., Nguyen W., Sivapatham R., Rane A., Andersen J.K. (2023). Urolithin A reduces amyloid-beta load and improves cognitive deficits uncorrelated with plaque burden in a mouse model of Alzheimer’s disease. Geroscience.

[56] Liu S., D’Amico D., Shankland E., Bhayana S., Garcia J.M., Aebischer P., Rinsch C., Singh A., Marcinek D.J. (2022). Effect of Urolithin A Supplementation on Muscle Endurance and Mitochondrial Health in Older Adults: A Randomized Clinical Trial. JAMA Netw. Open.

[57] Huang J.R., Zhang M.H., Chen Y.J., Sun Y.L., Gao Z.M., Li Z.J., Zhang G.P., Qin Y., Dai X.Y., Yu X.Y. (2023). Urolithin A ameliorates obesity-induced metabolic cardiomyopathy in mice via mitophagy activation. Acta Pharmacol. Sin..

[58] Cui G.H., Chen W.Q., Shen Z.Y. (2018). Urolithin A shows anti-atherosclerotic activity via activation of class B scavenger receptor and activation of Nef2 signaling pathway. Pharmacol. Rep..

[59] Tang L., Mo Y., Li Y., Zhong Y., He S., Zhang Y., Tang Y., Fu S., Wang X., Chen A. (2017). Urolithin A alleviates myocardial ischemia/reperfusion injury via PI3K/Akt pathway. Biochem. Biophys. Res. Commun..

[60] Nishimoto Y., Fujisawa K., Ukawa Y., Kudoh M., Funahashi K., Kishimoto Y., Fukuda S. (2022). Effect of urolithin A on the improvement of vascular endothelial function depends on the gut microbiota. Front. Nutr..

[61] Xia B., Shi X.C., Xie B.C., Zhu M.Q., Chen Y., Chu X.Y., Cai G.H., Liu M., Yang S.Z., Mitchell G.A. (2020). Urolithin A exerts antiobesity effects through enhancing adipose tissue thermogenesis in mice. PLoS Biol..

[62] Yang J., Guo Y., Henning S.M., Chan B., Long J., Zhong J., Acin-Perez R., Petcherski A., Shirihai O., Heber D. (2020). Ellagic Acid and Its Microbial Metabolite Urolithin A Alleviate Diet-Induced Insulin Resistance in Mice. Mol. Nutr. Food Res..

[63] Dahiya N.R., Chandrasekaran B., Kolluru V., Ankem M., Damodaran C., Vadhanam M.V. (2018). A natural molecule, urolithin A, downregulates androgen receptor activation and suppresses growth of prostate cancer. Mol. Carcinog..

[64] Wang Y., Qiu Z., Zhou B., Liu C., Ruan J., Yan Q., Liao J., Zhu F. (2015). In vitro antiproliferative and antioxidant effects of urolithin A, the colonic metabolite of ellagic acid, on hepatocellular carcinomas HepG2 cells. Toxicol. In Vitro.

[65] Cheng F., Dou J., Zhang Y., Wang X., Wei H., Zhang Z., Cao Y., Wu Z. (2021). Urolithin A Inhibits Epithelial-Mesenchymal Transition in Lung Cancer Cells via P53-Mdm2-Snail Pathway. Onco Targets Ther..

[66] Gimenez-Bastida J.A., Avila-Galvez M.A., Espin J.C., Gonzalez-Sarrias A. (2020). The gut microbiota metabolite urolithin A, but not other relevant urolithins, induces p53-dependent cellular senescence in human colon cancer cells. Food Chem. Toxicol..

[67] Norden E., Heiss E.H. (2019). Urolithin A gains in antiproliferative capacity by reducing the glycolytic potential via the p53/TIGAR axis in colon cancer cells. Carcinogenesis.

[68] Zhao W., Shi F., Guo Z., Zhao J., Song X., Yang H. (2018). Metabolite of ellagitannins, urolithin A induces autophagy and inhibits metastasis in human sw620 colorectal cancer cells. Mol. Carcinog..

[69] Kshirsagar S., Alvir R.V., Pradeepkiran J.A., Hindle A., Vijayan M., Ramasubramaniam B., Kumar S., Reddy A.P., Reddy P.H. (2022). A Combination Therapy of Urolithin A+EGCG Has Stronger Protective Effects than Single Drug Urolithin A in a Humanized Amyloid Beta Knockin Mice for Late-Onset Alzheimer’s Disease. Cells.

[70] Gong Q.Y., Cai L., Jing Y., Wang W., Yang D.X., Chen S.W., Tian H.L. (2022). Urolithin A alleviates blood-brain barrier disruption and attenuates neuronal apoptosis following traumatic brain injury in mice. Neural Regen. Res..

[71] Lin X.H., Ye X.J., Li Q.F., Gong Z., Cao X., Li J.H., Zhao S.T., Sun X.D., He X.S., Xuan A.G. (2020). Urolithin A Prevents Focal Cerebral Ischemic Injury via Attenuating Apoptosis and Neuroinflammation in Mice. Neuroscience.

[72] Qiu J., Chen Y., Zhuo J., Zhang L., Liu J., Wang B., Sun D., Yu S., Lou H. (2022). Urolithin A promotes mitophagy and suppresses NLRP3 inflammasome activation in lipopolysaccharide-induced BV2 microglial cells and MPTP-induced Parkinson’s disease model. Neuropharmacology.

[73] Liu J., Jiang J., Qiu J., Wang L., Zhuo J., Wang B., Sun D., Yu S., Lou H. (2022). Urolithin A protects dopaminergic neurons in experimental models of Parkinson’s disease by promoting mitochondrial biogenesis through the SIRT1/PGC-1alpha signaling pathway. Food Funct..

[74] Singh R., Chandrashekharappa S., Vemula P.K., Haribabu B., Jala V.R. (2020). Microbial Metabolite Urolithin B Inhibits Recombinant Human Monoamine Oxidase A Enzyme. Metabolites.

[75] Cortes-Martin A., Selma M.V., Tomas-Barberan F.A., Gonzalez-Sarrias A., Espin J.C. (2020). Where to Look into the Puzzle of Polyphenols and Health? The Postbiotics and Gut Microbiota Associated with Human Metabotypes. Mol. Nutr. Food Res..

[76] Avila-Galvez M.A., Gonzalez-Sarrias A., Espin J.C. (2018). In Vitro Research on Dietary Polyphenols and Health: A Call of Caution and a Guide on How To Proceed. J. Agric. Food Chem..

[77] Bookheimer S.Y., Renner B.A., Ekstrom A., Li Z., Henning S.M., Brown J.A., Jones M., Moody T., Small G.W. (2013). Pomegranate juice augments memory and FMRI activity in middle-aged and older adults with mild memory complaints. Evid. Based Complement. Altern. Med..

[78] Kaplan A., Zelicha H., Yaskolka Meir A., Rinott E., Tsaban G., Levakov G., Prager O., Salti M., Yovell Y., Ofer J. (2022). The effect of a high-polyphenol Mediterranean diet (Green-MED) combined with physical activity on age-related brain atrophy: The Dietary Intervention Randomized Controlled Trial Polyphenols Unprocessed Study (DIRECT PLUS). Am. J. Clin. Nutr..

[79] Kujawska M., Jourdes M., Witucki L., Karazniewicz-Lada M., Szulc M., Gorska A., Mikolajczak P.L., Teissedre P.L., Jodynis-Liebert J. (2021). Pomegranate Juice Ameliorates Dopamine Release and Behavioral Deficits in a Rat Model of Parkinson’s Disease. Brain Sci..

[80] Tan S., Tong W.H., Vyas A. (2020). Urolithin-A attenuates neurotoxoplasmosis and alters innate response towards predator odor. Brain Behav. Immun. Health.

[81] Jayatunga D.P.W., Hone E., Khaira H., Lunelli T., Singh H., Guillemin G.J., Fernando B., Garg M.L., Verdile G., Martins R.N. (2021). Therapeutic Potential of Mitophagy-Inducing Microflora Metabolite, Urolithin A for Alzheimer’s Disease. Nutrients.

[82] Jia F., Fellner A., Kumar K.R. (2022). Monogenic Parkinson’s Disease: Genotype, Phenotype, Pathophysiology, and Genetic Testing. Genes.

[83] Postuma R.B., Berg D., Stern M., Poewe W., Olanow C.W., Oertel W., Obeso J., Marek K., Litvan I., Lang A.E. (2015). MDS clinical diagnostic criteria for Parkinson’s disease. Mov. Disord..

[84] Simon D.K., Tanner C.M., Brundin P. (2020). Parkinson Disease Epidemiology, Pathology, Genetics, and Pathophysiology. Clin. Geriatr. Med..

[85] Yoo S.M., Jung Y.K. (2018). A Molecular Approach to Mitophagy and Mitochondrial Dynamics. Mol. Cells.

[86] Kelley N., Jeltema D., Duan Y., He Y. (2019). The NLRP3 Inflammasome: An Overview of Mechanisms of Activation and Regulation. Int. J. Mol. Sci..

[87] Gordon R., Albornoz E.A., Christie D.C., Langley M.R., Kumar V., Mantovani S., Robertson A.A.B., Butler M.S., Rowe D.B., O’Neill L.A. (2018). Inflammasome inhibition prevents alpha-synuclein pathology and dopaminergic neurodegeneration in mice. Sci. Transl. Med..

[88] Han X., Xu T., Fang Q., Zhang H., Yue L., Hu G., Sun L. (2021). Quercetin hinders microglial activation to alleviate neurotoxicity via the interplay between NLRP3 inflammasome and mitophagy. Redox Biol..

[89] Zhou Y., Wang S., Li Y., Yu S., Zhao Y. (2017). SIRT1/PGC-1alpha Signaling Promotes Mitochondrial Functional Recovery and Reduces Apoptosis after Intracerebral Hemorrhage in Rats. Front. Mol. Neurosci..

[90] Mudo G., Makela J., Di Liberto V., Tselykh T.V., Olivieri M., Piepponen P., Eriksson O., Malkia A., Bonomo A., Kairisalo M. (2012). Transgenic expression and activation of PGC-1alpha protect dopaminergic neurons in the MPTP mouse model of Parkinson’s disease. Cell Mol. Life Sci..

[91] Ng J.H.Y., Andersen J. (2022). Urolithin A: Gut-Brain Dietary Intervention in Parkinson’s Disease. Innov. Aging.

[92] Rezaee S., Jahromy M.H. (2018). Potential Effects of Pomegranate Juice in Attenuating LID in Mice Model of Parkinson Disease. Pharmacogn. J..

[93] Bar-Ya’akov I., Tian L., Amir R., Holland D. (2019). Primary Metabolites, Anthocyanins, and Hydrolyzable Tannins in the Pomegranate Fruit. Front. Plant Sci..

[94] Diaz-Mula H.M., Tomas-Barberan F.A., Garcia-Villalba R. (2019). Pomegranate Fruit and Juice (cv. Mollar), Rich in Ellagitannins and Anthocyanins, Also Provide a Significant Content of a Wide Range of Proanthocyanidins. J. Agric. Food Chem..

[95] Bellesia A., Verzelloni E., Tagliazucchi D. (2015). Pomegranate ellagitannins inhibit alpha-glucosidase activity in vitro and reduce starch digestibility under simulated gastro-intestinal conditions. Int. J. Food Sci. Nutr..

[96] Aichinger G., Stevanoska M., Beekmann K., Sturla S.J. (2023). Physiologically-based pharmacokinetic modeling of the postbiotic supplement urolithin A predicts its bioavailability is orders of magnitudes lower than concentrations that induce toxicity, but also neuroprotective effects. Mol. Nutr. Food Res..

[97] Yuan T., Ma H., Liu W., Niesen D.B., Shah N., Crews R., Rose K.N., Vattem D.A., Seeram N.P. (2016). Pomegranate’s Neuroprotective Effects against Alzheimer’s Disease Are Mediated by Urolithins, Its Ellagitannin-Gut Microbial Derived Metabolites. ACS Chem. Neurosci..

[98] Emami Kazemabad M.J., Asgari Toni S., Tizro N., Dadkhah P.A., Amani H., Akhavan Rezayat S., Sheikh Z., Mohammadi M., Alijanzadeh D., Alimohammadi F. (2022). Pharmacotherapeutic potential of pomegranate in age-related neurological disorders. Front. Aging Neurosci..

[99] Zaitone S.A., Abo-Elmatty D.M., Shaalan A.A. (2012). Acetyl-L-carnitine and α-lipoic acid affect rotenone-induced damage in nigral dopaminergic neurons of rat brain, implication for Parkinson’s disease therapy. Pharmacol. Biochem. Behav..

[100] Khan E., Hasan I., Haque M.E. (2023). Parkinson’s Disease: Exploring Different Animal Model Systems. Int. J. Mol. Sci..

[101] Guérin T., Waterlot C., Lipka E., Gervois P., Bulteel D., Betrancourt D., Moignard C., Nica A.S., Furman C., Ghinet A. (2021). Ecocatalysed Hurtley reaction: Synthesis of urolithin derivatives as new potential RAGE antagonists with anti-ageing properties. Sustain. Chem. Pharm..

[102] Siderowf A., Concha-Marambio L., Lafontant D.E., Farris C.M., Ma Y., Urenia P.A., Nguyen H., Alcalay R.N., Chahine L.M., Foroud T. (2023). Assessment of heterogeneity among participants in the Parkinson’s Progression Markers Initiative cohort using alpha-synuclein seed amplification: A cross-sectional study. Lancet Neurol..

[103] Dubey A.K., Chaudhry S.K., Singh H.B., Gupta V.K., Kaushik A. (2022). Perspectives on nano-nutraceuticals to manage pre and post COVID-19 infections. Biotechnol. Rep..

[104] Kumar R., Aadil K.R., Mondal K., Mishra Y.K., Oupicky D., Ramakrishna S., Kaushik A. (2022). Neurodegenerative disorders management: State-of-art and prospects of nano-biotechnology. Crit. Rev. Biotechnol..

[105] Romo-Vaquero M., Fernández-Villalba E., Gil-Martinez A.-L., Cuenca-Bermejo L., Espín J.C., Herrero M.T., Selma M.V. (2022). Urolithins: Potential biomarkers of gut dysbiosis and disease stage in Parkinson’s patients. Food Funct..

